# Primary Human Chondrocytes Affected by Cigarette Smoke—Therapeutic Challenges

**DOI:** 10.3390/ijms21051901

**Published:** 2020-03-10

**Authors:** Tao Chen, Sabrina Ehnert, Gauri Tendulkar, Sheng Zhu, Christian Arnscheidt, Romina H. Aspera-Werz, Andreas K. Nussler

**Affiliations:** Siegfried Weller Research Institute, Department of Trauma and Reconstructive Surgery, Eberhard Karls University Tübingen, BG Trauma Center Tübingen, 72076 Tübingen, Germany; zzuchentao@yahoo.com (T.C.); sabrina.ehnert@gmail.com (S.E.); gauritendulkar01@gmail.com (G.T.); zhusheng8686@gmail.com (S.Z.); carnscheidt@bgu-tuebingen.de (C.A.); rominaaspera@hotmail.com (R.H.A.-W.)

**Keywords:** cigarette smoke, osteoarthritis (OA), cartilage, chondrocyte, reactive oxygen species (ROS), dexamethasone, hyaluronic acid, diclofenac, acetaminophen

## Abstract

Although several researchers have attested deleterious effects of smoking to the musculoskeletal system, the association between smoking and the onset of osteoarthritis (OA) remains unclear. Here, we investigate the effect of cigarette smoke extract (CSE) on primary human chondrocytes. The present study demonstrates that physiological concentrations of CSE (0.1%–10%) inhibit the viability, proliferation, and matrix formation of chondrocytes in a dose- and time-dependent manner. Significant amounts of free radicals were generated by 10% of CSE and led to cell death. A clinical dosage (4 mg/mL) of dexamethasone (Dex) showed toxic effects on chondrocytes, and the long-time treatment by lower doses (4–400 μg/mL) induced hypertrophic changes in the chondrocytes. To substitute Dex, diclofenac (Dic, 1 μg/mL) and acetaminophen (Ace, 10 μg/mL) were tested and did not worsen the metabolic activity of CSE-exposed chondrocytes. Hyaluronic acid (HA, 5 mg/mL) combined with Dic or Ace significantly inhibited the oxidative stress and enhanced the viability and matrix formation of CSE-exposed chondrocytes. This study shows for the first time that CSE mediates the disruption of cartilage through inducing cell death by increasing oxidative stress, and that this effect is fortified by Dex. The deleterious effects of CSE on chondrocytes could be reversed by treatment with HA combined with first-line analgesic/anti-inflammatory agents.

## 1. Introduction

Cigarette smoke is one of the leading causes of preventable deaths in the world, jeopardizing the duration and quality of life. It is known that over 5000 harmful and toxic chemical compounds, as well as free radicals like reactive oxygen species (ROS) are contained in cigarette smoke [1,2]. Several in vitro and in vivo studies have proven the deleterious effects of cigarette smoke on the musculoskeletal system [3,4,5]. Continuous inhalation of cigarette smoke leads to surgical complications and prolonged hospital stays [5].

Osteoarthritis (OA), is one of the leading disabilities that affects millions of people in the world. It is well established that several environmental risk factors, such as obesity, injury, and occupational stress, are involved in the pathogenesis of OA [6,7]. Limited studies have evaluated the relationship between cigarette smoke and symptomatic OA, and the results are controversial [8,9]. The mechanisms by which smoking induces cartilage damage remain unclear. Chondrocytes are exclusively found in articular cartilage. They are capable of secreting extracellular matrix (ECM) to maintain the function and integrity of articular cartilage [10]. Some studies have claimed that nicotine, the physiological active component in cigarettes, increases proliferation, as well as mRNA and protein levels of type II collagen in chondrocytes of both healthy and OA patients [11,12]. However, cigarette smoke contains a large number of toxic and carcinogenic chemicals, such as carbon monoxide, polycyclic aromatic hydrocarbons, and hydrogen cyanide [13]. To our knowledge, no one has yet examined the direct effects of smoking on primary human chondrocytes or investigated the possible underlying mechanism.

Chondrocytes are normally in a quiescent condition and proliferation only occurs once cells are activated. Therefore, the survival and death of chondrocytes are vital for the preservation of articular cartilage [14]. It has been proposed that chondrocyte cell death occurs and engages in the development of OA, and is closely linked to the integrity of the cartilage matrix [15]. It is well-established that ROS are chemical constituents of cigarette smoke, crucial for producing adverse effects in human [16]. Increased oxidative stress could stimulate death signaling pathways to promote chondrocyte cell death and compromise cartilage integrity [17]. Therefore, it is assumed that ROS production caused by cigarette smoke leads to chondrocyte cell death and consequently to cartilage damage.

Nowadays, the intra-articular (IA) injection of hyaluronic acid (HA) and corticosteroids (CSs) are widely used in the treatment of OA [18,19]. HA is a high molecular weight glycosaminoglycan that is a component of the synovial fluid and ECM of articular cartilage in normal and OA patients [20]. In the synovial fluid of OA patients, the molecular size and concentration of HA are decreased [20]. The IA injection of HA is extensively applied and recommended in most guidelines as a viscosupplement for patients with OA [21,22]. Moreover, preliminary research has demonstrated that HA provides various biochemical and biological benefits for the chondrocytes, including chondro-protection [23], the scavenging of ROS [24], and the inhibition of inflammation [25]. CS injections in OA knees have been used to relieve pain and inhibit inflammation for many years and the efficacy has been assessed in a large number of studies [26,27]. Nevertheless, prolonged exposure to CSs may result in negative effects and accelerate OA progression [28]. Song et al. reported that glucocorticoids could suppress growth, as well as the gene expression and protein level of Sox9, type II collagen, aggrecan in human chondrocytes [29].

Although oral analgesic/anti-inflammatory agents, such as acetaminophen (Ace) and non-steroidal anti-inflammatory drugs (NSAIDs), are essential drugs for the pain management of OA patients, their severe side effects limit their application [18]. Recently, local IA injections of Ace and NSAIDs have been established, which have had strong anti-inflammatory effects in in vivo studies [30,31]. Therefore, IA injections of Ace or NSAIDs could be an alternative to alleviate joint-associated pain and inflammation in OA patients.

Thus, the aim of our study was to study the effects of cigarette smoke extract (CSE) on primary human chondrocytes, and furthermore, to investigate the possible mechanism by which CSE affects the chondrocytes viability and function. Lastly, to investigate whether pharmacologic treatments with dexamethasone (Dex) (representative for CSs), HA, Ace and diclofenac (Dic) (representative for NSAIDs) is beneficial to the chondrocytes impaired by CSE.

## 2. Results

### 2.1. CSE Exposure Inhibited the Viability, Proliferation, and Matrix Formation of Primary Human Chondrocytes in a Dose-Dependent Manner

**Figure 1:**
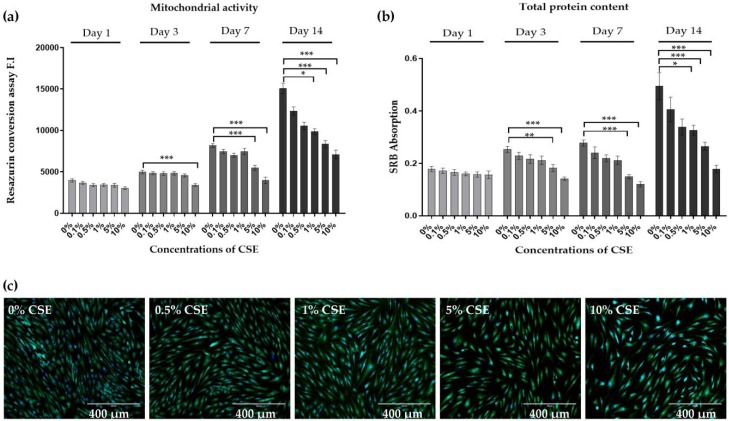
Cigarette smoke extract (CSE) exposure inhibited the viability and proliferation of primary human chondrocytes. Cells (*N* = 3, *n* = 3) were cultured in medium containing 0%, 0.1%, 0.5%, 1%, 5%, and 10% CSE. On day 1, 3, 7, and 14 chondrocytes (**a**) mitochondrial activity was determined by Resazurin conversion and (**b**) total protein content was determined by sulforhodamine B (SRB) staining, respectively. (**c**) Calcein-AM (green) and Hoechst 33,342 (blue) were used to show living cells on day 7, respectively. Results are presented as mean ± SEM. * *p* < 0.05, ** *p* < 0.01, *** *p* < 0.001 as compared to untreated controls.

To assess whether CSE influences the viability and proliferation of chondrocytes, primary human chondrocytes were exposed to different concentrations of CSE (0.1%, 0.5%, 1%, 5%, and 10%) every day and analyzed on days 1, 3, 7, and 14, respectively. The effects on the mitochondrial activity and total protein content, which are indirect indicators of viability and proliferation, were measured by resazurin conversion and Sulforhodamine B (SRB) staining, respectively (Figure 1a,b). The mitochondrial activity and total protein content of chondrocytes exposed to CSE were significantly affected in a dose- and time-dependent manner compared to untreated controls. On days 7 and 14, chondrocytes treated with 5% and 10% CSE showed a significant reduction in mitochondrial activity (*** *p* < 0.001) and total protein content (*** *p* < 0.001), respectively. Similarly, Calcein-AM staining showed a dose-dependent decline of cell viability by CSE when compared to controls (Figure 1c).

**Figure 2:**
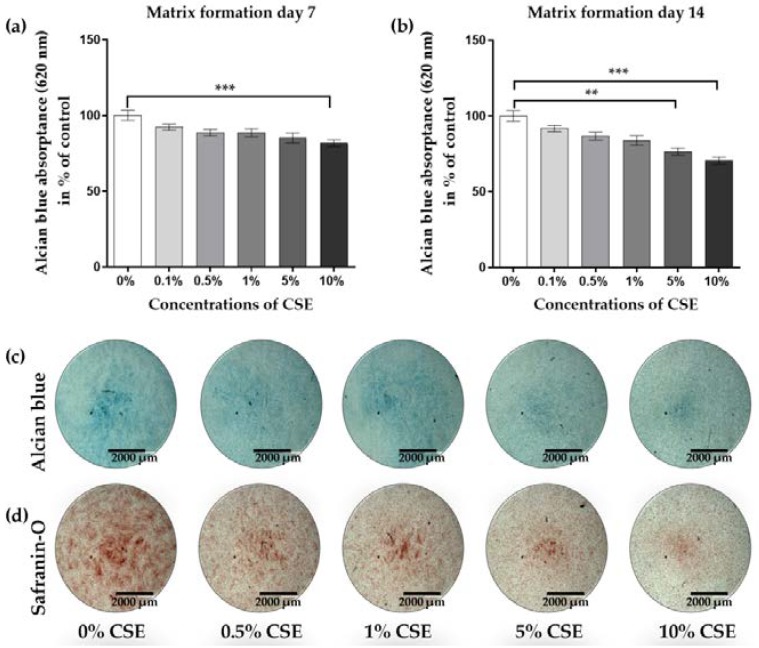
CSE exposure decreased the matrix formation of human primary chondrocytes. Cells (*N* = 3, *n* = 3) were cultured in a medium containing 0%, 0.1%, 0.5%, 1%, 5%, and 10% CSE. Matrix formation was determined quantitatively by resolving Alcian blue staining after growing in the medium with or without CSE for 7 (**a**) and 14 days (**b**), respectively. (**c**, **d**) Representative Alcian blue and Safranin-O stainings are shown to visualize matrix formation on day 7. Results are given as fold of control (untreated cells) and presented as mean ± SEM. ** *p* < 0.01, *** *p* < 0.001 as compared to untreated cells.

In order to determine whether CSE also affects the function of chondrocytes, Alcian blue and Safranin-O staining were performed to evaluate the expression of proteoglycans and type II collagen, which are the main components of the ECM from chondrocytes [10]. Matrix formation of CSE-exposed chondrocytes was dose-dependently decreased on day 7 (Figure 2a, 10%, *** *p* < 0.001) and day 14 (Figure 2b, 5%, ** *p* < 0.01 and 10%, *** *p* < 0.001 vs. untreated controls), respectively. Consistent with the quantitative analysis, Alcian blue and Safranin-O staining showed decreased blue (Figure 2c) and red (Figure 2d) stains on day 7, respectively. Based on these data, 10% CSE was selected for further experiments.

### 2.2. CSE Exposure Induced Cell Death by Increasing ROS Production

**Figure 3:**
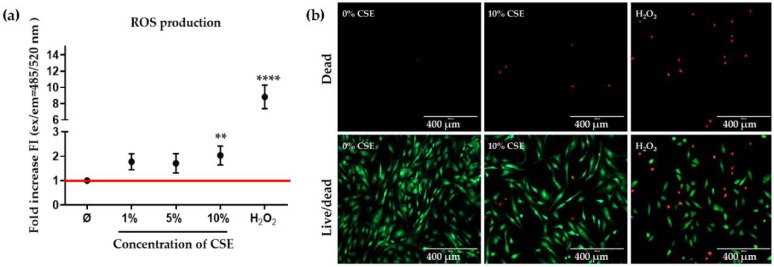
CSE exposure induced cell death by increasing ROS production. Cells (*N* ≥ 3, *n* ≥ 3) were cultured in a medium containing 0%, 1%, 5%, and 10% CSE or 0.01% H_2_O_2_. (**a**) Dichlorfluorescein-diacetate (DCFH-DA) assay was used to evaluate reactive oxygen species (ROS) production in primary human chondrocytes exposed to CSE, and 0.01% *v/v* H_2_O_2_ was used as a positive control. (**b**) Calcein-AM (green) and Ethidium homodimer (red) were administered to show living and dead cells on day 3, respectively, and 0.01% *v/v* H_2_O_2_ was used as a positive control. Results are given as fold of control (untreated group, represented in red line) and presented as mean ± SEM. ** *p* < 0.01, **** *p* < 0.0001 as compared to control group.

It is well known that ROS are chemical constituents of cigarette smoke that are crucial to produce adverse effects in humans [16]. Compelling studies have indicated that chondrocyte cell death occurs and contributes to OA development and that ROS are among the main factors that induce cell death [15,17]. The production of ROS was measured in CSE-exposed chondrocyte cultures using the dichlorfluorescein-diacetate (DCFH-DA) assay. The highest ROS production was seen when exposing chondrocytes to 10% CSE compared to untreated cells, (Figure 3a, ** *p* < 0.01). As the positive control we chose 0.01% hydrogen peroxide, demonstrating the cell death of chondrocytes (Figure 3b) by increased ROS levels.

### 2.3. Clinical Dose of Dex Reduced Viability of Primary Human Chondrocytes

**Figure 4:**
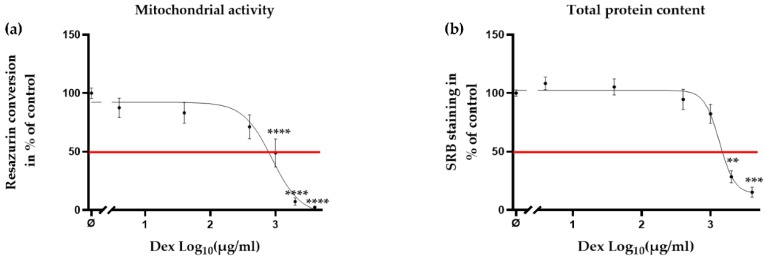
Clinical dose of Dex reduced the viability of primary human chondrocytes. Cells (*N* ≥ 3, *n* ≥ 3) were incubated with Dex (4–4000 μg/mL) without CSE. After 24 h of treatment, the viability of the cells was measured by (**a**) mitochondrial activity (resazurin conversion) and (**b**) total protein content (SRB staining), respectively. Results are presented as mean ± SEM. Half maximal effective concentration (EC 50) is presented in red line. ** *p* < 0.01, *** *p* < 0.001, **** *p* < 0.001 as compared to untreated cells.

The IA injection of CSs, such as Dex, is widely used in relieving pain and inflammation of OA patients [26]. CSs are both immune-suppressive and anti-inflammatory [27]. Furthermore, CSs have been reported to inhibit ROS formation [32]. Nevertheless, preliminary studies suggested destructive effects of Dex on the viability and proliferation of chondrocytes [28,33]. In order to determine the concentrations of Dex that are non-toxic to the cells, chondrocytes were incubated with Dex (4–4000 μg/mL) for 24 h. It is noteworthy, that the clinical doses used to treat OA are up to 4000 μg/mL [34,35]. Mitochondrial activity (Figure 4a, ≥1000 μg/mL, **** *p* < 0.0001) and total protein content (Figure 4b, ≥2000 μg/mL, ** *p* < 0.01 and *** *p* < 0.001) decreased dose dependently when chondrocytes were treated with Dex, suggesting that the clinical dose of Dex is detrimental to chondrocytes.

### 2.4. Effects of HA and Decreased Concentrations of Dex on the CSE-Impaired Primary Human Chondrocytes

**Figure 5:**
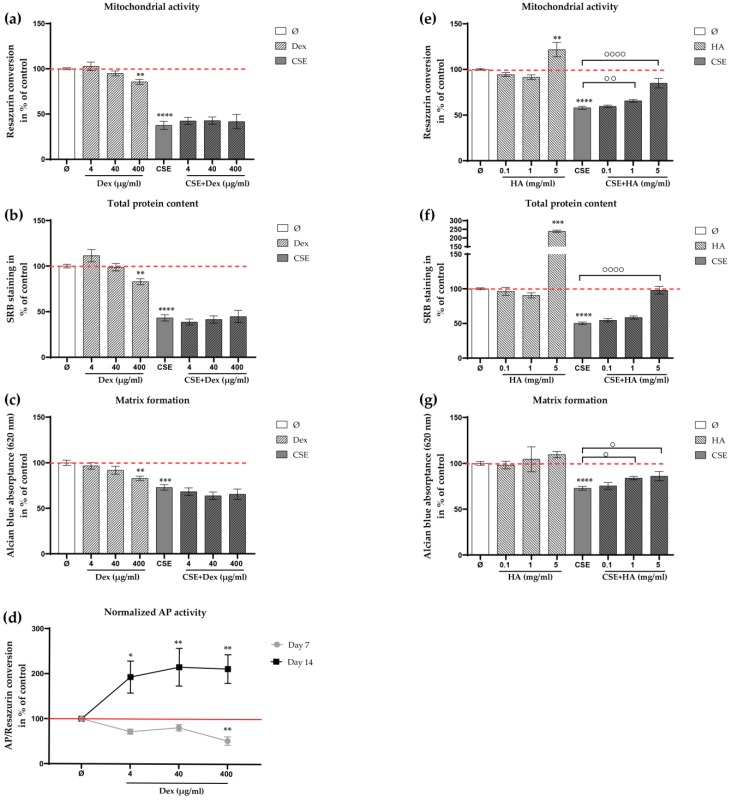
Effects of hyaluronic acid (HA) and decreased concentrations of dexamethasone (Dex) on CSE-impaired primary human chondrocytes. Cells (*N* ≥ 3, *n* ≥ 3) were incubated with Dex (4, 40, and 400 μg/mL) or HA (0.1, 1, and 5 mg/mL) with or without 10% CSE. After 7 days of treatment, viability of the cells was measured by (**a**, **e**) mitochondrial activity (resazurin conversion) and (**b**, **f**) total protein content (SRB staining). (**c**, **g**) Matrix formation of cells was evaluated by quantifying Alcian blue staining. (**d**) AP activity of chondrocytes in the presence of different concentrations of Dex on day 7 and day 14. Results are given as fold change of control (untreated cells, represented in red line or red dotted line) and presented as mean ± SEM. * *p* < 0.05, ** *p* < 0.01, *** *p* < 0.001, **** *p* < 0.0001 vs. the control group, ° *p* < 0.05, °° *p* < 0.01, °°°° *p* < 0.0001compared to cells treated with CSE alone.

The IA injection of HA is thought to provide viscosity to joints, as the molecular weight and concentration of HA decrease in OA patients [20]. Additionally, HA is thought to provide diverse biochemical and biological benefits to chondrocytes, including chondroprotection [23], the scavenging of ROS [24], and the inhibition of inflammation [25]. Therefore, in the next step, we investigated whether CSE-impaired chondrocytes could be rescued by HA or decreased concentrations of Dex. Based on the above results (Figure 5a,b), three concentrations of Dex (4, 40, and 400 μg/mL) were tested in the following experiments. Mitochondrial activity, total protein content, and matrix formation showed a dose-dependent decrease (Figure 5a–c, 400 μg/mL, ** *p* < 0.01) when the chondrocytes were treated with Dex for 7 days. Interestingly, no significant difference was observed between CSE-impaired chondrocytes and cells with additional Dex treatment on day 7. Alkaline phosphatase (AP) activity (a marker of hypertrophic chondrocytes) increased significantly (Figure 5d, ≥4 μg/mL, * *p* < 0.05, ** *p* < 0.01, and ** *p* < 0.01, respectively) when the cells were treated with Dex for 14 days, indicating that Dex may modify chondrocyte morphology after long-time exposure. On the contrary, HA increased significantly the mitochondrial activity (Figure 5e, ≥1 mg/mL, ** *p* < 0.01 and **** *p*< 0.0001) and the total protein content (Figure 5f, 5 mg/mL, **** *p* < 0.0001) of CSE-impaired chondrocytes after 7 days. In addition, the accumulation of matrix formation by chondrocytes was significantly enhanced (Figure 5g, ≥1 mg/mL, * *p* < 0.05) when compared to cells treated with CSE alone on day 7.

### 2.5. Ace and Dic Did not Augment Adverse the Effects of CSE on Primary Human Chondrocytes

**Figure 6:**
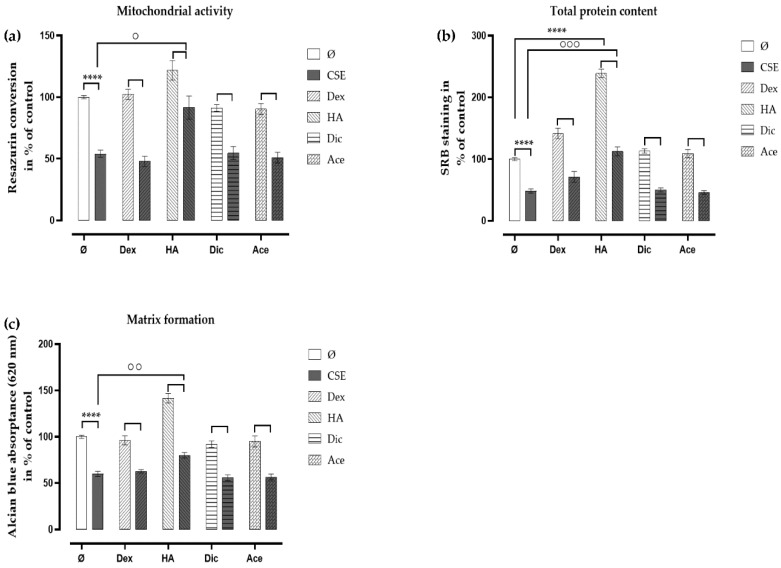
Diclofenac (Dic) and acetaminophen (Ace) had no additional adverse effect on CSE-impaired primary human chondrocytes. Cells (*N* = 3, *n* = 3) were incubated with Dex (4 μg/mL), HA (5 mg/mL), Dic (1 μg/mL), or Ace (10 μg/mL) with or without 10% CSE. After 7 days of treatment, the viability of the cells was measured by (**a**) mitochondrial activity (resazurin conversion) and (**b**) total protein content (SRB staining). (**c**) Matrix formation was quantified by resolving the Alcian blue staining. Results are given as fold of control (untreated cells) and presented as mean ± SEM. * *p* < 0.05, **** *p* < 0.0001 compared to untreated cells, ° *p* < 0.05, °° *p* < 0.01, °°° *p* < 0.001 compared to cells treated with CSE alone.

Due to the severe side effects of the oral administration of analgesic/anti-inflammatory agents, the IA injection of these drugs appears to be a promising alternative, and many in vivo studies have confirmed the anti-inflammatory effects of the IA injection of Ace and NSAIDs [30,31]. In order to evaluate their effects on chondrocytes, therapeutic concentrations of Ace (10 μg/mL) and Dic (1 μg/mL) were applied daily on the chondrocytes with or without CSE. On day 7, both Ace and Dic did not further affect the mitochondrial activity (Figure 6a) of chondrocytes exposed to CSE, which was consistent with the results obtained by total protein content (Figure 6b). Matrix formation was not affected by Ace and Dic as well (Figure 6c).

### 2.6. Analgesic/Anti-Inflammatory Agents in Combination with HA Rescued the CSE-Impaired Primary Human Chondrocytes

**Figure 7:**
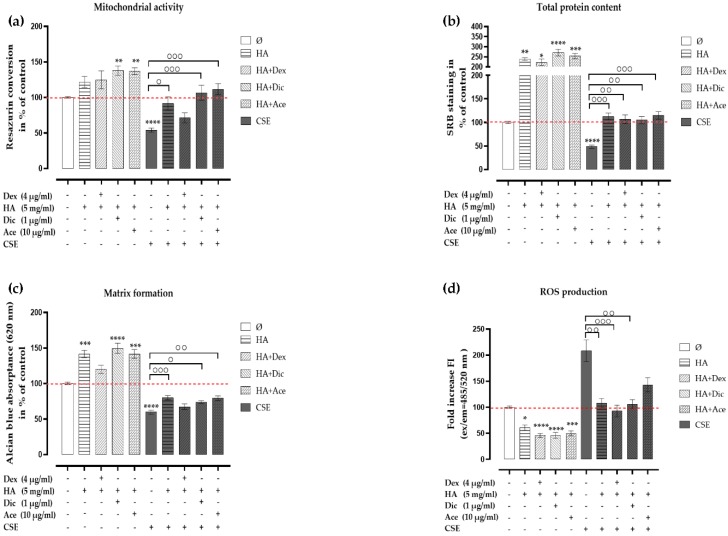
Analgesic/anti-inflammatory agents in combination with HA rescued CSE-impaired primary human chondrocytes. Cells (*N* ≥ 3, *n* = 3) were incubated with Dex (4 μg/mL), Dic (1 μg/mL), or Ace (10 μg/mL) in combination with HA (5 mg/mL) with or without 10% CSE. After 7 days of treatment, the viability of the cells was measured by (**a**) mitochondrial activity (resazurin conversion) and (**b**) total protein content (SRB staining). (**c**). Matrix formation of the cells was quantified by resolving the Alcian blue staining. (**d**). DCFH-DA assay was used to evaluate ROS production in primary human chondrocytes co-incubated with or without 10% CSE and treatments, and 0.01% *v/v* H_2_O_2_ served as a positive control. Results are given as fold change of control (untreated cells, represented in red dotted line) and presented as mean ± SEM. * *p* < 0.05, ** *p* < 0.01, *** *p* < 0.001, **** *p* < 0.0001 compared to untreated cells, ° *p* < 0.05, °° *p* < 0.01, °°° *p* < 0.001 compared to CSE treated cells.

However, Dex, Dic, and Ace are normally used for pain relief and inflammation inhibition. In OA patients, not only pain relief and the suppression of inflammation should be addressed, but also cartilage repair. In order to investigate the effects of HA in combination with analgesic/anti-inflammatory agents (Dex, Dic, or Ace) on chondrocytes impaired by CSE, cells were cultured in a medium supplemented either with HA (5 mg/mL) alone or in combination with Dex (4 μg/mL), Dic (1 μg/mL), or Ace (10 μg/mL). After 7 days of co-incubation, HA combined with Dic or Ace significantly increased the mitochondrial activity (Figure 7a, HA+Dic,*** *p*< 0.001 and HA+Ace, *** *p* < 0.001), as well as matrix formation (Figure 7c, HA+Dic, * *p* < 0.05 and HA+Ace, ** *p* < 0.01) in CSE-exposed chondrocytes compared with chondrocytes exposed to 10% CSE alone. An increased trend in the mitochondrial activity and matrix formation was observed when HA treatment was combined with Dex. All HA combinatory treatments significantly increased the total protein content (Figure 7b, HA+Dex, ** *p* < 0.01, HA+Dic, ** *p* < 0.01, and HA+Ace, *** *p* < 0.001) compared with chondrocytes exposed to 10% CSE alone. ROS production was significantly inhibited in HA treatment and all HA combinatory treatments (Figure 7d, HA+Dex, ** *p*< 0.01, HA+Dic, ** *p*< 0.01 and HA+Ace, *** *p*< 0.001), suggesting that HA and HA combinatory treatments rescued the CSE-impaired chondrocytes by means of reducing ROS formation.

## 3. Discussion

Cigarette smoke is a toxic and carcinogenic mixture of over 5000 chemicals, including nicotine, tar, heavy metals, and hydrogen cyanide [1]. Several in vitro, in vivo, and clinical studies have shown a positive association between cigarette consumption and musculoskeletal disorders [3,5]. Sporadic studies have evaluated the relationship between cigarette smoke and symptomatic OA, however, these studies showed conflicting results [8,9,36]. Besides, these studies may be in part or not conclusive, as they are based on radiographic images or the total knee replacement outcome. It was reported that physiological concentrations of nicotine may increase the proliferation of chondrocytes, and upregulated mRNA and protein level of collagen II [11,12]. Nevertheless, nicotine is not the one and only toxin found in cigarettes and the half-life of nicotine is around 2 h [37]. Toxins contained in cigarettes have been shown to induce oxidative stress [2], inflammatory responses [38], or hypoxia [39], which all can damage cartilage. Recently, we reported that CSE negatively affects the migration, proliferation, and chondrogenic differentiation of mesenchymal stem cells through impairing the transforming growth factor- β (TGF-β) signaling [40]. However, the effects of cigarette smoke on primary human chondrocytes remain unclear. Moreover, a number of researchers have attested that the chondrogenic capacity is not significantly affected by OA, and OA chondrocytes are suitable for the tissue engineering of articular cartilage [41,42,43]. Therefore, we chose human OA chondrocytes for our study and investigated the effects of primary human chondrocytes exposed to physiological concentrations of CSE every day.

We observed a significant inhibition of viability, proliferation, and function in CSE-exposed primary human chondrocytes. We subsequently pursued an investigation into how CSE negatively affected the cells. It is known that ROS in, or induced by, cigarette smoke are crucial risk factors for damaging human health. Kamceva et al. reported that the number of cigarettes smoked has a key role in increasing oxidative stress and thereby reducing anti-oxidant defense [2]. Aspera-Werz et al. reported that although nicotine and cotinine did not directly induce ROS, they impaired the anti-oxidative defense mechanisms within mesenchymal stem cells by inhibiting anti-oxidative enzyme activity [44]. In our study, ROS production was generated significantly when chondrocytes were exposed to 10% CSE, which is in line with smoking approximately 20 cigarettes (1 pack) a day [40]. As chondrocytes are normally in a quiescent condition and only proliferate once activated, the survival and death of chondrocytes are vital for the preservation of articular cartilage [14]. Chondrocyte cell death occurs and favors the development of OA and is closely linked to the integrity of the cartilage matrix [15]. It has been suggested that enhanced oxidative stress could stimulate death signaling pathways, such as the p38 signaling pathway, to trigger chondrocyte cell death and consequently compromise cartilage integrity [17]. In our study, we observed that the treatment of chondrocytes with 10% CSE or 0.01% H_2_O_2_ equally caused a significant reduction of cell growth and induction of cell death. Chondrocyte cell death may occur through apoptosis [45], necrosis [46], autophagy [47], or a combination of these processes [48]. Therefore, further work is needed to determine which mode of cell death is executed in CSE-induced cell death.

The IA injection of CSs, such as Dex, may provide pain relief and reduce inflammation in patients with knee OA [21]. Previous studies have shown the beneficial effects of Dex on inhibiting the accumulation of inflammatory cells and inflammatory cytokines, such as interleukin-1β, in the affected joint [27]. Moreover, it is widely accepted that Dex exerts immune-suppressive and anti-inflammatory effects by inhibiting ROS production [32]. Although injections of steroids are effective in relieving joint inflammation and its symptoms, they should be used with caution due to side effects, including the systemic effects of steroids, infectious arthritis, and cartilage damage [49]. Preliminary studies reported that Dex may inhibit chondrocyte growth and reduce the expression of Sox-9, type II collagen, and aggrecan in a time- and dose-dependent manner [29,50]. Our results indicated that clinical concentrations of Dex are toxic to chondrocytes, while lower concentrations (4–400 μg/mL) seem to be non-toxic, when no additional CSE is applied. These results are in agreement with the findings of Busse et al. [51], showing that lower concentrations of Dex have less detrimental effects on chondrocytes compared to clinical concentrations. In addition, the work of Stewart and colleagues reported that treatment with Dex stimulated AP activity and AP mRNA of equine mesenchymal stem cells and might, therefore, induce the cells to differentiate toward bone [52]. Similarly, our study found that AP activity increased significantly when chondrocytes were treated with Dex for 14 days, suggesting that Dex modifies chondrocyte morphology and function after long-time exposure. Therefore, it is necessary to balance the efficacy, dose, and treatment duration of Dex when it is used in patients with OA, especially when they are smokers.

HA, as a high molecular weight glycosaminoglycan, is used with IA injections to improve the elasticity and viscosity of the synovial fluid in OA joints [20,53]. Researchers have demonstrated beneficial effects of HA on the chondrocytes, including chondroprotection [23], the scavenging of ROS [24], and the inhibition of inflammation [25]. For IA injection, usually 2 to 3 mL of HA with a concentration of 10 mg/mL are applied to the joint [54], which has about 0.5–4.0 mL of synovial fluid with a pH of between 7 and 8 [55]. Based on that, the dilution of HA is approximately 1:2, which is consistent with the concentrations used in our study. In these doses, HA had stimulatory effects on the metabolic activity of CSE-impaired chondrocytes, particularly at high doses (5.0 mg/mL). In the study of Akmal et al., low doses of HA (0.1 and 1.0 mg/mL) significantly increased DNA content, sulphated glycosaminoglycan, and hydroxyproline synthesis in bovine articular chondrocytes [23]. In our study, a higher concentration of HA might have been required as cells were additionally exposed to CSE, which has been reported to down-regulate the expression of the CD44 receptor and to limit the interaction of chondrocytes with their surrounding ECM [56], which might decrease the cells’ response toward HA.

The chronic nature of OA requires pharmacological treatment over a prolonged timespan. Owing to limited distribution to the inflamed tissues [57] and the severe side effects/risks of oral analgesic or anti-inflammatory agents [58,59], IA injections of NSAIDs or Ace appear a promising attempt at relief pain in OA joints [30,31,60]. In order to assess the feasibility of the IA injection of Ace and NSAIDs, the concentrations of drugs used in our study were equivalent to the therapeutic concentrations observed in blood plasma [61]. Additionally, the short half-life of Ace (2–3 h) [62] and Dic (1.2–2 h) [63] would require frequent injections to maintain therapeutic levels. Therefore, we treated chondrocytes with these drugs every day in our experimental setup. Our study suggested that therapeutic concentrations of Ace and Dic had no additional adverse effect on the viability and function of CSE-impaired primary human chondrocytes. Similarly, Blot et al. found that, in human moderate and severe OA cartilage, the metabolic balance of proteoglycan and HA are unaffected in the presence of 0.3–3 μg/mL Dic [64]. Other in vivo studies have shown analgesic and anti-inflammatory effects of IA injections of Dic [65] and Ace [30], approving their feasibility for IA injection as a substitute treatment in patients with OA. Based on the data presented here, this is of special importance for smokers with OA, as this might reduce the possible side effects expected from IA injections of Dex in these patients.

It is noteworthy that OA is a whole joint disease, involving the loss of articular cartilage, synovial inflammation, and subchondral bone remodeling [18]. Thus, not only pain relief and suppression of inflammation should be addressed, but also cartilage repair. Recent reports have investigated the combined effects of HA and anti-inflammatory drugs (CSs or NSAIDs) [66,67] in order to develop more effective OA treatments. We evaluated the combined effects of HA with low concentrations of Dex and therapeutic concentrations of Dic or Ace on CSE-impaired chondrocytes. The results revealed that treatment combinations activated the metabolic activity of chondrocytes under smoking conditions but had no synergistic effects compared with HA treatment alone. Yu et al. demonstrated that HA reduced H_2_O_2_ and O^2−^ in the synovial fluid of OA patients, thus suppressing H_2_O_2_-induced cell death in human OA chondrocytes [24]. In our study, ROS production was also inhibited by means of HA treatment and all HA combinatory treatments, suggesting anti-oxidative effects of HA by scavenging free radicals.

In addition, chondrocytes and immune cells are involved in catabolic and inflammatory processes during OA, suggesting the detrimental results of in vitro conditions are likely to be, at least in part, attenuated in animals and human patients. For the future, it remains to be elucidated whether the changes observed in the metabolism of chondrocytes would also occur in cartilage in vivo. Moreover, as the frequency and dose required of the drugs depend on various factors in humans, further in vivo investigations need to be assessed prior to clinical application.

## 4. Materials and Methods

Acetaminophen (Ace), diclofenac (Dic), hyaluronic acid (HA), and dexamethasone (Dex) were obtained from Sigma-Aldrich (A5000-100G, D6899-10G, 53747, D2915-100MG; Darmstadt, Germany).

### 4.1. Ethics Statement

All human samples were obtained in accordance with the Ethics Commission of the Medical Faculty of the Eberhard-Karls University, University Hospital Tübingen (653/2018BO2, dated on 20 September 2018) and the 1964 Helsinki declaration and its later revisions. A consent form was obtained from all participants in the study.

### 4.2. Generation of Cigarette Smoke Extract (CSE)

CSE was freshly prepared before each experiment. Briefly, two commercial cigarettes (Marlboro, Philip Morris, New York City, USA), which contained 0.8 mg nicotine and 10 mg tar each, were continuously combusted through a standard gas washing bottle (Lenz Laborglas GmbH & Co.KG, Wertheim, Germany). The smoke was bubbled into a 50 mL pre-warmed Dulbecco’s Modified Eagle’s Medium (DMEM) (Sigma-Aldrich, Darmstadt, Germany), at an average speed of 95 bubbles/min. The concentration of CSE was determined and normalized by its optical density at λ = 320 nm (OD_320_), and an OD_320_ of 0.7 was considered 100% CSE. Before using the fresh CSE, it was filtered through a sterile filter with a pore size of 0.22 μm. Then the CSE was further diluted (0.1%, 0.5%, 1%, 5%, and 10%) with chondrocyte culture medium. The CSE concentrations corresponded to exposures associated with smoking from 0.01 pack (0.1%) to 1 pack (10%) of cigarettes/day [3].

### 4.3. Isolation and Culture of Human Primary Chondrocytes

Primary human chondrocytes were isolated from the femoral condyles or tibia plateaus of patients who had received a total knee replacement. The isolation of primary human chondrocytes was performed as described before [68]. Briefly, the cartilage (donor number *N* = 14; 10 males and 4 females; age = 69.64 ± 8.82 years) was cut into pieces and thoroughly washed with phosphate-buffered saline (PBS) (without Ca^2+^/Mg^2+^, Merck, Darmstadt, Germany). Afterward, the pieces were digested by collagenase (1500 U/mL, GIBCO, Darmstadt, Germany) at 37 °C overnight, then, the supernatant was centrifuged in order to remove the collagenase. Thereafter, cells obtained were expanded in culture medium (DMEM/Ham’s F12 (1:1) supplemented with 10% Fetal Calf Serum (FCS, GIBCO, Darmstadt, Germany), 1% penicillin/streptomycin (Merck, Darmstadt, Germany) and 50 µM l-ascorbate-2-phosphate (Merck, Darmstadt, Germany) at 37 °C and 5% CO_2_. For all experiments, a medium with or without CSE was changed every day and cultures were investigated at 3, 7, and 14 days.

### 4.4. Resazurin Conversion Assay

Resazurin conversion assay was employed to assess cell viability. Briefly, cells were incubated with 0.0025% *w/v* Resazurin solution (Sigma-Aldrich, Darmstadt, Germany) (in PBS) for 30 min at 37 °C, the fluorescence of converted Resorufin was measured (ex/em = 540/590 nm) with a plate reader (Omega, Germany), as described previously [69].

### 4.5. Sulforhodamine B (SRB) Staining

Total protein content was determined by SRB staining. After fixation with ethanol (1 h at –20 °C), cells were stained with 0.4% *w*/*v* SRB (Sigma-Aldrich, Darmstadt, Germany) (in 1% *v*/*v* acetic acid) for 30 min at room temperature (RT). Cells were washed 4–5 times with 1% acetic acid to remove unbound SRB. Bound SRB was resolved with 10 mM unbuffered TRIS solution (pH = 10.5, Sigma-Aldrich, Darmstadt, Germany). The resulting absorption (λ = 565 nm/OD_565_) was determined with a plate reader, as previously described [69].

### 4.6. Live/Dead Staining with Calcein-AM and Ethidium Homodimer

Living cells were determined by intra-cellular esterase activity staining using Calcein-AM (green fluorescence, Sigma-Aldrich, Darmstadt, Germany) and dead cells using Ethidium homodimer (red fluorescence, Sigma-Aldrich, Darmstadt, Germany) staining. Nuclei were visualized by Hoechst 33,342 (blue fluorescence, Sigma-Aldrich, Darmstadt, Germany). Briefly, chondrocyte cultures with or without CSE were washed three times with PBS and incubated with Calcein-AM (2 µM), Ethidium homodimer (4 µM), and Hoechst 33,342 (1 mg/mL) at RT for 30 min. Fluorescent images were captured (Epifluorescence: EVOS FL, life technologies, Darmstadt, Germany) following washing with PBS [70].

### 4.7. Histochemical Analysis of ECM Production

In order to assess the production of glycosaminoglycans (GAGs) and type II collagen, Alcian blue and Safranin-O stainings were performed as recently published [68]. Briefly, after removing the cell culture medium, cells were washed with PBS, then fixed with 4% *v*/*v* formaldehyde (Carl Roth, Karlsruhe, Germany) for 30 min and washed again twice with PBS. Afterwards, 1% *w*/*v* Alcian blue (pH = 1.0, Carl Roth, Karlsruhe, Germany) and 0.1% *w*/*v* Safranin-O (Carl Roth, Karlsruhe, Germany) staining solution were added to stain the cells at RT for 30 min. Subsequently, unbounded dye solution was removed with distilled water. The stained cells were imaged using the EVOS FL microscope. For quantitative analyses, Alcian blue-stained cultures were extracted with 6 M guanidine HCl (Sigma-Aldrich, Darmstadt, Germany) in distilled water at RT overnight [71]. The optical density of the extracted dye was measured at λ = 620 nm (OD_620_) with a plate reader.

### 4.8. Reactive Oxygen Species (ROS) Production Analysis with DCFH-DA Assay

Prior to experiments, cells were washed twice with PBS. Subsequently, they were incubated with 10 μM 2′, 7′-dichlorfluorescein-diacetate (DCFH-DA, Sigma-Aldrich, Darmstadt, Germany) in serum-free culture medium for 30 min at 37 °C. After washing the cells twice with PBS, cells were stimulated with CSE. The fluorescence intensity, representing reactive oxygen species (ROS) levels, was quantified using a plate reader (ex/em = 485/520 nm) [3].

### 4.9. Alkaline Phosphatase (AP) Activity Assay

AP activity was measured as a marker of hypertrophic chondrocytes [72]. Briefly, cells were incubated with AP reaction buffer (0.2% *w/v* 4-nitrophenyl-phosphate, 50 mM glycine, 1 mM MgCl_2_, 100 mM TRIS, and pH = 10.5) for 60 min at 37 °C. Formed 4-nitrophenol was determined photometrically (λ = 405 nm/OD_405_) as described in [3] and normalized to relative cell numbers by Resazurin conversion. Changes in the AP activity are displayed relative to untreated cells.

### 4.10. Statistics

Graph Pad Prism software (version 8.0, El Camino Real, CA, USA) was used to analyze data. Data are presented as mean ± SEM (*N* ≥ 3, *n* ≥ 3) for each group. The differences between multiple groups were estimated by the Kruskal–Wallis H test followed by Dunn’s multiple comparison test. The Mann-Whitney *U*-test (2-sided) was used to compare two single groups with each other. *p* < 0.05 was considered to be the minimum level of statistical significance.

## 5. Conclusions

In conclusion, our study demonstrated for the first time that CSE inhibits the viability, proliferation, and matrix formation of primary human chondrocytes. Furthermore, it demonstrated that CSE induces cell death by the accumulation of ROS. Low doses and a short-time exposure of Dex is correlated with the maintenance of cell viability and cartilage morphology, while clinical doses and a long-time exposure have rather detrimental effects on chondrocytes. In contrast, therapeutic concentrations of Dic and Ace had no additional adverse effects on the metabolic activity of chondrocytes exposed to CSE, suggesting that IA injections of Ace and NSAIDs are promising substitutes for Dex in the treatment of symptomatic OA, especially in smokers. HA and HA combined with low doses of Dex, or therapeutic doses of Dic, and Ace proved to be efficient in scavenging free radicals, as well as in protecting chondrocytes from toxic CSE.

## Abbreviations

Ace	Acetaminophen
CSE	Cigarette smoke extract
OA	Osteoarthritis
ROS	Reactive oxygen species
ECM	Extracellular matrix
IA	Intra-articular
HA	Hyaluronic acid
CSs	Corticosteroids
NSAID	Non-steroidal anti-inflammatory drug
OD	Optical density
PBS	Phosphate-buffered saline
RT	Room temperature
SRB	Sulforhodamine B
GAG	Glycosaminoglycan
DCFH-DA	Dichlorfluorescein-diacetate
AP	Alkaline Phosphatase
H_2_O_2_	Hydrogen peroxide
TGF-β	Transforming growth factor β
PAHs	Polycyclic aromatic hydrocarbons
Dic	Diclofenac
Dex	Dexamethasone
EC 50	Half maximal effective concentration
DMEM	Dulbecco’s Modified Eagle’s Medium
FCS	Fetal Calf Serum
